# A System of Medicine by Many Writers

**Published:** 1908-03

**Authors:** 


### Reviews of Books.

**A System of Medicine by many writers.** Edited by THOMAS CLIFFORD ALLBUTT, M.A., M.D., and HUMPHRY DAVY ROLLESTON, M.A., M.D. Volume II., Part I. Pp. xii., 1087. 1906. Part II. Pp. xvi., 1055. 1907. Volume III. Pp. xii., 1040. 1907. London: Macmillan and Co., Limited.

The arrangement of Volume II. is much altered. Tropical diseases and internal parasites go elsewhere, and rheumatic fever has been brought in. Two hundred pages are allotted to a general pathology of infection by Professor Ritchie, which forms a welcome guide to the overwhelming literature of immunity and the action of bacteria on the body. There is also considerable advantage in having collected together for comparison full accounts of the various antitoxins, antivenenes and opsonins, instead of discussing them under the several diseases, such as diphtheria, tuberculosis, &c. Thus the interesting and detailed account of opsonins shows their relation to the different theories of phagocytosis and immunity. Ritchie concludes his long article by pointing to the foundation for an extended knowledge of the normal interaction of the elements or cells of the body, which is given by this study of their resistance to bacteria. For this resistance, after all, is only an application of the normal processes of nutrition and digestion.

A separate paper is devoted to the pathology of streptothrix infections, while that of protozoa is discussed in another volume. Hutchinson is thus relieved from giving detailed accounts of the spirochæta of syphilis, though he appears fairly satisfied as to its causal relations to the disease. It may be noted that he is still unconvinced as to the proofs of transmission of syphilis to a third generation.

In the pathology of scarlatina, Dudgeon discusses the rival organisms of Klein, Class and Mallory, and thinks that the main question centres around the streptococci, about which unfortunately all is in confusion.

One of the most interesting articles is that of McVail on the protection afforded by vaccination, with the results of recent investigations.

The theory of ptomaine poisoning has been vigorously pruned, and the symptoms are now in most cases referred to the rapid action of pathogenic organisms, such as the B. enteritidis. Phosphorus poisoning is well described, but the result shows how contradictory are the theories of its pathology. Oliver has improved his article on plumbism, and added a too brief account of the granular degeneration of the red cells, but here and in other articles we could wish for more editorial influence in the direction of conciseness and classification. On page 904, line 13 it would seem that a misprint of "observed" for "prolonged" has slipped in.

The size and scope of the second part of this volume, and the fact that the editors have seen fit to depart from their original scheme, in so far as to issue a volume of their *System of Medicine* entirely devoted to this group of diseases, sufficiently indicate the rapid growth of what is practically a new branch of medicine, namely tropical medicine.

In a sense, knowledge, more or less complete, of most of the diseases included under tropical medicine has existed as a matter of ancient history; what is new lies in their investigation by modern methods, which in no branch of medicine have been productive of more fruitful results. These methods of investigation, giving us for the first time a scientific insight into the real nature and modes of origin of tropical diseases, have completely transformed the subject. That we are only upon the threshold of this great branch of medicine is evident, and every year may be expected to mark a fresh advance. It is impossible in the limits of our space to give any adequate account of the whole contents of this volume; it must suffice to say that it is likely to be the standard work of reference on the subject for some years to come. Throughout the book the subject dealt with has been entrusted to competent hands, and the reader may confidently rely on finding information, which is trustworthy and embodies recent work. A feature of the book are the special articles or, as the editors justly entitle them, monographs on the biology of the numerous animal parasites which produce or convey tropical diseases. These articles are by well-recognised authorities, and are liberally and effectively illustrated. Prof. Minchin deals with the protozoa, Mr. F. V. Theobald with mosquitoes, Mr. E. E. Austen with blood-sucking flies, Mr. R. I. Pocock with ticks, and Mr. A. E. Shipley has adapted to modern nomenclature Sir Patrick Manson's article on worms. To workers in the tropics these articles must prove of great value, and will be further read with interest by all who wish to keep abreast of medical science. Nothing is more remarkable than the contrast in this one respect between the important part now played by biological studies in medicine and the very small bearing that such studies appeared to have upon it at so short an interval of time as thirty years ago.

It is not only to medical men practising in the tropics that this book will be useful, for in these days of rapid and easy intercommunication between all parts of the world, and of the growing importance of our colonial empire, a practitioner in England may be at any time called upon to diagnose and treat some disease imported from beyond the seas. For instance, last winter a case of kala-azar was admitted into one of the Bristol hospitals. If he can take down this book from his shelves, he will get from it just the kind of help he requires, and obtain not merely a smattering, but a truly scientific account of the disease he may have to treat. The rapid transit of the present day has indeed brought fresh problems to be solved by the practitioner in the tropics, for as appears in several articles in this volume, facilities for travel aid in the conveyance of infective diseases directly by man, or indirectly by parasites, in a way formerly unknown.

The third volume has also been largely rewritten. Dr. A. E. Garrod contributes new articles on rheumatoid arthritis, osteoarthritis, spondylitis and the joint lesions in infective diseases. The distinction drawn between the first and second diseases is quite definite. The cases in middle-aged women, commonly called rheumatoid, are here classed as osteoarthritis, and similar instances are said occasionally to occur in children. He recognises the self-limited course of rheumatoid arthritis in spite of its tendency to relapses, and gives an interesting account of recent work on the bacteriology, confessing that it is as yet impossible to say whether Schuller's villous arthritis is really distinct. There clearly is a monoarticular type sometimes limited to one finger, but more often seen in the knee or shoulder, and it seems difficult to imagine that this can be an early stage of ordinary rheumatoid. On the other hand, we miss any definite account of the type where every joint is affected and anchylosed. Still this may possibly be due to individual peculiarities. The article, and especially the remarks on treatment, are well abreast of our present knowledge. Some note of Latham's spinal blisters might, however, have been added.

Rose Bradford revises the late Sir W. Roberts' paper on gout, adding some account of the purin bodies generally, and performs a similar task for the late Dr. Ralfe's diabetes insipidus.

One of the most perfect and charming articles is the last work of the late Professor Dreschfeld on ulcer of the stomach, which gives a most interesting account of recent work on this difficult subject, of the different lines of treatment recommended, the various complications and the theories of its pathology. He points out the need of improving the general nutrition of the body as rapidly as possible, if we are to hope for a complete cure without the disheartening relapses which attend the ordinary methods.

Treves's articles on appendicitis, obstruction, visceroptosis and acute peritonitis, as well as Allingham's rectal diseases, have been more or less rewritten by other authors. Lockwood's account of appendicitis is a mine of information on the pathology and complications of this disease, and his minor clinical hints, such as the deceptive symptoms seen in gouty patients and the importance of a careful rectal examination, are well worth remembering.

The revised chapter on the colon epitomises the recent discussions on ulcerative colitis and asylum dysentery, but the author gives his reasons for separating them both from true dysentery. We must not omit to notice the contributions of Acland on subphrenic and other peritoneal abscesses, and the new articles on bacteriology by Andrewes and Slater. On the whole, great labour has been expended to incorporate all important additions to our knowledge in the subjects treated of in this volume.

